# Acculturation of hygiene norms among immigrants to Sweden

**DOI:** 10.3389/fpsyg.2023.975361

**Published:** 2023-02-06

**Authors:** Joel Krueger, Kimmo Eriksson, Isabela Hazin, Andrey Tibajev, Pontus Strimling

**Affiliations:** ^1^Institute for Futures Studies, Stockholm, Sweden; ^2^School for Education, Culture and Communication, Mälardalen University, Västerås, Sweden; ^3^Department of Women’s and Children’s Health, Uppsala University, Uppsala, Sweden; ^4^Department of Management and Engineering, The Institute for Analytical Sociology, Linköping University, Linköping, Sweden

**Keywords:** acculturation, hygiene, social norms, social sanctions, immigrants, Sweden

## Abstract

Hygiene norms in Sweden are generally loose compared to most other countries. Does this looseness affect the hygiene norms among people who immigrate to Sweden from other countries? In a study of hygiene norms among immigrants to Sweden, the change in the physical environment and material living conditions, acculturation to Swedish culture and norms, and selection effects were all expected to lead immigrant hygiene norms to be closer to Swedish looseness. However, in a sample of 447 immigrants from 12 different countries, immigrants reported hygiene norms that were even *stricter* than those found in their countries of origin. We propose an explanation based on a combination of uncertainty about prevailing hygiene norms and the social risk and stigma associated with being perceived as unhygienic. We conclude that acculturation processes may rely on mechanisms that are domain specific.

## Introduction

In the past few decades there has been a rapid rise in global migration, and more people than ever now live outside their country of origin (United Nations Department of Economic and Social Affairs, 2020). There are several reasons why people migrate, including for work or education, to reunite with family, and to escape conflict and persecution. With large disparities in wealth, global tension, and more frequent natural disasters, migration rates are expected to stay elevated within the foreseeable future (McAuliffe and Triandafyllidou, 2021). This makes acculturation, how individuals adjust to a new cultural environment, a hot topic. Here we are exclusively concerned with the descriptive aspect of acculturation. Thus, our object of study is *how* immigrants adapt to the majority culture and not whether they *should* adapt.

Acculturation may be studied in any behavioral domain in which there are cultural differences. Our focus here is injunctive norms about hygiene. Injunctive norms are beliefs about how one ought to behave, as distinct from how people actually behave. Norms are important for individual and group identity and set up shared expectations for appropriate behaviors (Terry et al., 1999; Hogg and Reid, 2006). The way injunctive norms prescribe or prohibit certain behaviors also make them powerful drivers of behavioral change (Reno et al., 1993; Carey et al., 2007; Smith and Louis, 2008).

Hygiene norms have long been studied in sociology as an example of how norms change over time (Elias, 1978). Specific hygiene norms are socially learned and vary between cultures, sometimes combining ideas about purity and contamination to take on symbolic and ritualistic connotations (Miller, 1998; Preston and Ritter, 2012). Poor hygiene is often a source of shame, social disapproval, and stigma (Haidt et al., 1997; Brewis et al., 2019). Such negative judgments are mediated by feelings of disgust, a core human emotion that is thought to have evolved to trigger disease-avoidant behaviors (Oaten et al., 2009; Curtis et al., 2011; Schaller and Park, 2011). Perceived disease threats have also been shown to be associated with xenophobia, conformity, and in-group bias (Faulkner et al., 2004; Schaller and Neuberg, 2012; Wu and Chang, 2012), which adds complexity as well as relevance to investigating immigrant acculturation of hygiene norms.

Hygiene is of great importance for global public health and reducing the spread of disease (Prüss-Ustün et al., 2019; World Health Organization, 2022). Despite this, the literature on acculturation with respect to hygiene is small. It consists mostly of studies focused on how to improve health literacy and hygiene standards of certain immigrant or minority groups (McDonald et al., 2008; Lee, 2016; Davis, 2021). Here, we ask a different question: What happens to people’s hygiene norms when they move to a country where hygiene norms are looser than in their country of origin? Specifically, we focus on immigration to Sweden, a country where a recent international survey has shown that the strictness of injunctive norms, that is, beliefs about how one ought to behave, are relatively loose regarding handwashing, spitting, and toothbrushing (Eriksson et al., 2021a). In our study, we repeat this survey among recent immigrants to Sweden, allowing us to compare the strictness of hygiene norms among immigrants to the corresponding norms in the immigrants’ countries of origin and the broader Swedish population.

### Background

There are at least three types of reasons to expect the hygiene norms of immigrants to differ from norms in their countries of origin. First, material living conditions may affect the strictness of hygiene norms. If living conditions differ between Sweden and their countries of origin, immigrants may readily adopt hygiene norms that are better suited to Swedish conditions. Second, immigrant hygiene norms may change as part of a larger cultural adaptation to Swedish norms and behavioral expectations. Third, the hygiene norms of immigrants may differ from their country of origin due to selection effects. We discuss each of these possibilities in turn.

#### Material living conditions and disease threat

The three types of hygiene norms assessed in this study—handwashing, toothbrushing, and refraining from spitting—are all quite low-tech. Nonetheless, several reviews of interventions promoting handwashing highlight that communities in low-income countries often lack access to soap and water (Dreibelbis et al., 2014; Wolf et al., 2019). Inadequate drinking water and poor hygiene and sanitation practices are known to be causally implicated in the higher disease burden in these countries (Freeman et al., 2014; Prüss-Ustün et al., 2019). In addition to extending access to soap and water, many interventions in low- and middle-income countries focus on how to induce norm changes to promote better hygiene practices (Dreibelbis et al., 2013; McMichael, 2019; Wolf et al., 2019). Weak hygiene norms in vulnerable communities are likely due to the bi-directionality of *descriptive* norms, beliefs about what people do, and *injunctive* norms, beliefs about what people ought to do (Eriksson et al., 2015). For instance, if nobody can wash their hands with soap and water, it is unlikely that the injunctive norm that one should do so will be maintained.

However, the only study, to date, directly investigating how hygiene norms vary across countries found that Sweden, a country with high material living conditions, has looser hygiene norms than most of the world (Eriksson et al., 2021a). The same study also found hygiene norms to be stricter in countries with higher levels of perceived threat of disease. Sweden has a very low historical prevalence of infectious diseases and the lowest levels of perceived threat of disease in the world (Murray and Schaller, 2010; Eriksson et al., 2021a). Thus, the higher disease burden in low-income countries may, in part, explain why lower-income countries were found to have higher levels of perceived disease threat as well as stricter hygiene norms than Sweden. Though there is no necessary relationship between actual and perceived disease threat, things like visibly dirty water and smelliness from bad sanitation may make the threat of disease salient, which could influence perceived disease threat (Culpepper et al., 2018). Thus, the living environment in Sweden might lower perceptions about disease threat and lead to corresponding adjustments in hygiene norm strictness.

#### Acculturation

*Acculturation* refers to the process of social, psychological, and cultural change that results from being immersed in a new cultural environment. Immigrants face the challenge of how to deal with differences in social norms and cultural practices between their native culture and their receiving society. Assimilation is one possibility, that is immigrants gradually identifying less with their native culture while absorbing more and more of the majority/dominant culture. However, there are other ways to navigate a new cultural environment. Berry (1992, 1997) proposed that identification with each of the two cultures should be viewed as separate dimensions: whether immigrants consider their cultural identity and customs valuable and worth retaining, and whether they consider relations with the larger society valuable enough to be sought. Positive or negative attitudes toward these two dimensions yield four possible acculturation strategies: *integration* (+/+), *assimilation* (−/+), *separation* (+/−), and *marginalization* (−/−). A person with an integration strategy aims to maintain their native culture while also adopting the culture of their new society. A person adopting an assimilation strategy aims to give up their native culture and fully take on the cultural values, norms, and traditions of their new society. A separation strategy involves wishing to preserve one’s native culture while avoiding interactions with the new society. Finally, marginalization involves little interest or involvement in either culture (Sam and Berry, 2010).

A body of empirical studies has shown that nearly all immigrant groups prefer integration and are most negative toward marginalization (Van Oudenhoven et al., 1998; Neto, 2002; Sam and Berry, 2010). However, the context of their own immigration situation, such as cultural differences, social conditions, and historical intergroup relations, can force immigrants to adopt non-preferred acculturation strategies (Roccas et al., 2000; Phillimore, 2011). Recognizing this complexity, Navas et al.’s (2005) Relative Extension Acculturation Model (RAEM) differentiates between *ideal* strategies, that immigrants would use if they could, and the *real* strategies, that immigrants say they practice in their new society.

The RAEM also predicts that a person’s real strategies will differ for different domains of social life. More symbolic and ideological domains, like values and religion, are more central to people’s cultural identities, and cultural change in these areas requires a fundamental readaptation of the individual or group. Thus, Navas et al. (2005) predict that immigrants are more likely to opt for a strategy close to *separation* in more symbolic and ideological domains. In contrast, people are expected to view it as less important to maintain their original culture in more materialistic and instrumental domains, like work and consumption habits, and are thus predicted to be more likely to opt for *integrating* or even *assimilating* strategies in such cultural domains. A subsequent study provided support for these predictions: immigrants adopted an assimilation strategy for work and consumption habits, while a separation strategy was adopted for family, social relations, values, and religion (Navas et al., 2007). Similar results were reported in a study on Muslim immigrants to Western Europe, showing that immigrants adopted the work-related values of their new countrymen while maintaining their original values with regard to family and religion (Pettersson, 2007).

According to the RAEM framework, the real acculturation strategy applied to hygiene norms will, in part, depend on whether they are perceived as mostly materialistic and instrumental or symbolic and ideological (Navas et al., 2005). Though hygiene has symbolical connotations, the main function of hygiene norms is to promote healthy behaviors and prevent contamination and the spread of disease (Curtis et al., 2011). Consequently, culturally diffused information and recommendations from the host society may be more likely to affect hygiene norms than norms in more symbolic domains. Against this background, we expected the studied hygiene norms to be viewed as generally more instrumental and materialistic, and thus relatively susceptible to influence from the Swedish majority culture.

#### Selection effects

The aim of the present study is to investigate the cultural adjustment of immigrants. However, the survey data is cross-sectional, not longitudinal. We compare immigrant hygiene strictness with strictness in immigrants’ countries of origin. There is a risk that immigrants’ hygiene norms, already upon arrival in Sweden, were not representative of their countries of origin. Hygiene norms may vary between groups in the same country, and certain groups may also be more likely to migrate. People who wish to permanently move to another country are often young and highly educated (Esipova et al., 2011; Docquier et al., 2014). A study on migration intentions in Ghana, Senegal, Morocco, and Egypt found that the typical potential migrant was young, male, and displayed relatively modern values, operationalized as approval of unmarried women migrating (van Dalen et al., 2005). Sweden has the most individualistic and secular values in the world (Haerpfer et al., 2020), so voluntary migrants with more modern values may be particularly likely to choose Sweden as a destination country. A first step to eliminate selection effects is therefore to control for the effects of age, gender, education, and modern values when comparing hygiene norms between samples.

Selection effects would also arise if approval of Swedish hygiene norms were a common reason for choosing Sweden as a destination. But this is improbable for a couple of reasons. Immigrants are unlikely to know much about how hygiene norms compare between countries. Moreover, to the extent that migrants can choose their destination countries, factors that most influence their choices are things like social networks, family reunification, economic possibilities, and the belief that a country is democratic and likely to respect human rights and rule of law (Neumayer, 2004; Mallett and Hagen-Zanker, 2018; Crawley and Hagen-Zanker, 2019). Among such concerns, the strictness of hygiene norms is an unlikely priority.

#### Hypothesis

With regards to immigrants’ hygiene norm strictness, all three of the discussed factors—material living conditions and disease threat, acculturation, and selection effects—seem to work in the same direction. The material living conditions and low prevalence of disease in Sweden could shift immigrants’ hygiene strictness closer to that of Swedes. The same goes for immersion in the Swedish dominant culture, particularly assuming that the studied hygiene norms are more instrumental and materialistic than symbolic and ideological. We control for several potential selection effects, but if there are other ways in which immigrants are not representative of their countries of origin, this is likely to also be in the direction of being more similar to Swedes. Thus, our pre-registered hypothesis was that *the strictness of immigrant hygiene norms would be somewhere in between the strictness of their country of origin and the strictness of Swedes*.

## Materials and methods

A survey was conducted measuring hygiene norm strictness among recent immigrants to Sweden. The strictness in Sweden and among nationals in the immigrants’ countries of origin were previously surveyed as part of the International Study of Metanorms (ISMN; Eriksson et al., 2021b). Our hypothesis and approach to analysis were, in general terms, preregistered with AsPredicted.^1^

### Participants

Participants for the immigrant data collection were recruited from a participant pool created for an ongoing research project studying the values of immigrants to Sweden, the Swedish Immigrant Values Survey. Newly arrived immigrants are a known hard-to-survey population, with specific challenges including, e.g., incorrect sampling frames due to mobility and out-migration and high refusal rates caused by lower levels of literacy and issues with trust in anonymity (Reichel and Morales, 2017; Lynn et al., 2018). For the original survey, participants were recruited at Swedish for Immigrant schools across Sweden, answering the survey both with digital aids in classrooms and online. Swedish for Immigrants is a cost-free adult education offered to almost all newly arrived immigrants, making it an institutionalized point of entry into the Swedish society and one of the best possible recruitment sites for newly arrived immigrants. Comparisons between the values of the participants in the original survey and immigrants in population-wide surveys using probability sampling techniques, e.g., the World Values Survey, show that no major differences exist between the two (Tibajev et al., 2022). More information on the sampling, data, and content of the Swedish Immigrant Values Survey is available at Strimling and Vartanova (2022).

The participant pool consists of 3,364 immigrants from 129 different countries. Participation was compensated with a grocery store voucher worth 100 kr (∼12 USD). We preregistered two criteria for participation. First, we were only interested in participants from countries for which there is ISMN data. Second, we preregistered that we would only use countries from which there were at least 50 immigrants in the participant pool. However, for ethical reasons we did not store any personal data, and, therefore, had no way to selectively invite only individuals from countries of interest. Thus, an invitation to take the survey was sent out to the entire pool, but with information in the invitation concerning which countries were our primary interest. Response rates were 59% among participants from the targeted countries. Immigrants in our participation pool who no longer reside in Sweden were excluded, as well as participants who did not pass the attention check or did not complete all relevant items. A cut-off point was set to include countries with responses from more than 20 immigrants. A total of 12 countries met this cut-off: Brazil, China, Greece, India, Iran, Italy, Poland, Spain, Turkey, Ukraine, the United Kingdom, and the United States.

The ISMN used convenience samples of college students, aiming to recruit at least 200 students in 57 different countries. The main sampling strategy was complemented with data collected from non-student samples in 28 countries. Participants were recruited using a variety of methods, including email invitations, social media, in-class, face-to-face on campus, public notices and flyers, and using survey organizations. For more details, see Eriksson et al. (2021b).

The final sample of immigrants consisted of 447 participants (55% female, mean age 32.9). The final sample of Swedes, from the ISMN dataset, consisted of 203 participants (54% female, mean age 27.2). The final samples from immigrants’ countries of origin, also from the ISMN dataset, on average consisted of 414 participants (70% female, mean age 24.8), ranging from 222 participants in Iran to 997 in China.

The demographic profiles of immigrants to Sweden, nationals in the countries of origin, and Swedes are summarized in Table 1. For a summary of the demographic profiles for immigrants and nationals, separated by country of origin, see Supplementary Table 2.

**TABLE 1:** Description of sample characteristics for immigrants to Sweden, non-immigrant nationals in the countries of origin, and Swedes.

Sample	*N*	Gender	Age		Education		Modern values	
		(% female)	*M*	SD	*M*	SD	*M*	SD
Immigrants	447	55.03	32.87	7.59	2.83	0.43	0.83	0.24
Nationals	4963	70.34	25.23	10.04	2.66	0.63	0.67	0.25
Swedes	203	53.69	27.22	9.34	3.00	0.00	0.87	0.16

### Procedure

Participants in the immigrant sample were reached via email and completed the questionnaire online. Before completing the survey, all participants received written information about the research project and gave their informed consent. The questionnaire consisted of 30 items and took approximately 15 min to complete. In addition to items about demographics, hygiene norms, and modern values, the questionnaire included items about perceived cultural tightness and about meta norms concerning the appropriateness of various responses to norm violations (such as gossip, ostracism, or confrontation). The data on metanorms and cultural tightness were not used in the present study.

### Measures of hygiene norm strictness

Hygiene norm strictness was assessed with items concerning three different hygiene domains: handwashing, spitting, and toothbrushing. The measures were the same as previously used in the ISMN (Eriksson et al., 2021b).

#### Handwashing

To measure handwashing norms, participants were asked “In which situations do you think people should wash their hands?” with tick boxes for the following six situations: before eating, after eating, after defecating, after urinating, after coming home, and after shaking someone’s hand. In the original ISMN survey, these contexts were selected to represent common situations with relevance for handwashing. For each participant, the proportion of boxes ticked represented their individual handwashing strictness (theoretically varying between 0 and 1). Averaging across participants in each population yielded population measurements of handwashing strictness.

#### Spitting

To measure spitting norms, participants were asked “Where do you think it is not appropriate for people to spit?” with tick boxes for the following six locations: in the kitchen sink, on the sidewalk, on the kitchen floor, on a soccer field, in a public pool, and the forest. In the original ISMN survey, these contexts were selected to represent situations where spitting is common or a relevant hygiene concern. For each participant, the proportion of the boxes ticked represented their individual spitting strictness (theoretically varying between 0 and 1). Averaging across participants in each population yielded population measurements of spitting strictness.

#### Toothbrushing

To measure toothbrushing norms, participants were asked “How often do you think people should brush their teeth?”. In the original ISMN survey, the response options were taken from Maes et al. (2006), except that the high-end of their scale (“more than once a day”) was separated into two options (“two times a day” and “three times a day or more”). The response options were coded between 0 and 1: three times a day or more (coded as 1), two times a day (coded as 2/3), once a day (coded as 1/3), at least one time a week (coded as 0), less than one time a week (coded as 0), and never (coded as 0). The three options on the low end represented so few responses that they were coded together as a single category of low toothbrushing strictness. Averaging scores across participants in each population yielded population measurements of toothbrushing strictness, theoretically varying between 0 and 1.

### Control variables

Demographic questions included age, gender, modern values, and education. Modern values were measured by each participant’s average response to three items about how justifiable they find homosexuality, abortion, and divorce. Responses were recorded on a 10-point Likert scale (1–Never justifiable, 10–Always justifiable). The response options for education differed slightly between the immigrant survey and the ISMN survey. The response options for both surveys were translated to a three-level scale: 1 = no high school education, 2 = high school education, and 3 = higher education. For the immigrant survey, “less than high school degree” was coded as 1; “high school graduate” was coded 2; and “some college but no degree,” “Bachelor’s/Associate degree,” “Master’s degree,” and “Doctoral degree” were coded 3. For the ISMN survey, “incomplete compulsory school,” “compulsory school,” and “vocational training for manual work” were coded 1; “upper secondary education” and “post-secondary education” were coded 2; and “university education 3 years or more” was coded 3.

### Exclusion of items affected by COVID

Hygiene norm strictness was surveyed in many countries in 2019 as part of the ISMN (Eriksson et al., 2021b). This was before the COVID-19 pandemic. As outlined in the preregistration, the plan was to compare this data with data from a subsequent data collection on hygiene norm strictness among immigrants to Sweden. Unfortunately, the COVID-19 pandemic broke before the new data were collected in May 2021. During the pandemic, public health agencies emphasized the importance of good hand hygiene. Thus, certain hygiene norms are expected to have become stricter in response to COVID, which is a concern for comparisons between the two datasets.

The questions for handwashing and spitting each consist of six separate binary items, whereas toothbrushing frequency was measured with a single item. This means that, in total, hygiene strictness was assessed through 13 items. In a study still under review, the first wave of the ISMN was compared with a second wave (in 43 of the original 57 countries) that was conducted during the COVID pandemic in 2020. Only two hygiene items showed significant change since the pre-COVID study: norms about washing hands after coming home and washing hands after shaking hands had become much stricter across the globe. Consequently, these two norms were excluded from the analysis. No other hygiene norms showed significant change since the pre-COVID study.

### Statistical analysis

The mean strictness scores for handwashing, spitting, and toothbrushing were calculated separately for immigrants to Sweden, nationals in the countries of origin, and Swedes. Initial checks of the data revealed that the assumptions of normality and homogeneity of variance were violated. For this reason, proportional odds ordinal logistic regression was used to estimate mean strictness, adjusting for age, gender, and modern values. Wald tests were performed to test differences between the estimated means.

Adjusted mean strictness for handwashing, spitting, and toothbrushing was also estimated for each country separately. A chi-square test was run on the ordinal regression model to test whether the country of origin affected the difference in strictness between immigrants and their countries of origin and whether these differences differed between hygiene domains (handwashing, spitting, and toothbrushing).

The samples of nationals and immigrants were both dominated by highly educated participants, but the Swedish sample had only highly educated participants, which made controlling for education across samples impossible. To test the effects of education on the strictness of hygiene norms, we performed an additional analysis contrasting the strictness of participants with higher education with the strictness of participants without higher education across all 28 countries with non-student samples in the ISMN. Multiple linear regression controlling for country, age, gender, and modern values showed no effect of high education on norm strictness in any of the three hygiene domains. See Supplementary Figure 1.

In their analysis of hygiene norms in ISMN, Eriksson et al. (2021b) found that the 13 items loaded on two distinct factors, which did not align perfectly with the three hygiene domains (handwashing, spitting, and toothbrushing). We preregistered that we would analyze these two factors as distinct subscales in the current study. However, after excluding the two items affected by COVID, the relevance of the original factor decomposition is questionable. In the main article, we instead report results by domain, which has the added advantage of making the results easier to interpret. Readers interested in results per factor can refer to the Supplementary material.

## Results

**FIGURE 1:**
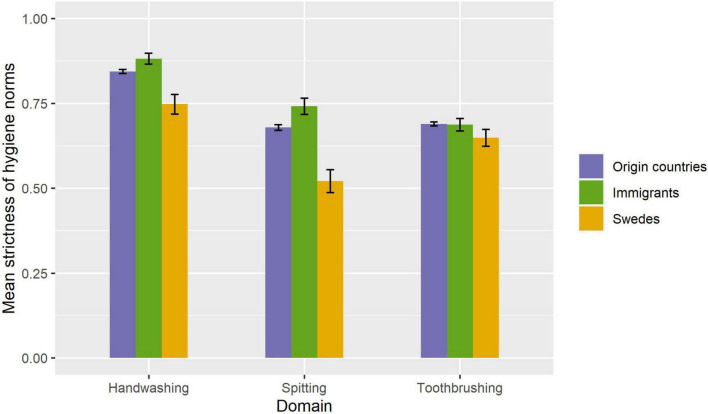
Estimated mean strictness of norms of immigrants to Sweden, nationals in immigrants’ countries of origin, and the broader Swedish population. Estimates were calculated using proportional odds ordinal logistic regression, adjusting for age, gender, and modern values. Error bars signify 95% confidence intervals.

The proportional odds ordinal logistic regression was used to estimate the mean strictness of hygiene norms, adjusting for age, gender, and modern values. The adjusted mean strictness for each group in each domain is shown in Figure 1.

The comparison between immigrants and nationals for toothbrushing was not significant. All other comparisons between groups were significant at the 0.05 significance level, See Table 2. Contrary to our hypothesis, immigrant hygiene strictness was not in between that of nationals and Swedes in any of the three hygiene domains. Instead, in both the handwashing and spitting domains, there was an effect in the opposite direction: immigrants had even stricter hygiene norms than nationals in their countries of origin.

**TABLE 2:** Differences in hygiene strictness between immigrants, non-immigrant nationals in the countries of origin, and Swedes.

Contrast	Effect	95% CI	*SE*	*Z*-statistic	p
**Immigrants-Nationals**					
Handwashing	0.44	[0.24, 0.65]	0.11	4.25	<0.001
Spitting	0.46	[0.28, 0.65]	0.09	4.89	<0.001
Toothbrushing	−0.03	[−0.26, 0.19]	0.11	0.23	0.819
**Immigrants-Swedes**					
Handwashing	1.41	[1.09, 1.73]	0.16	8.56	<0.001
Spitting	1.55	[1.27, 1.83]	0.15	10.68	<0.001
Toothbrushing	0.45	[0.10, 0.79]	0.18	2.54	0.011
**Nationals-Swedes**					
Handwashing	0.96	[0.70, 1.23]	0.14	7.03	<0.001
Spitting	1.10	[0.85, 1.32]	0.12	9.09	<0.001
Toothbrushing	0.47	[0.18, 0.76]	0.15	3.18	<0.001

Wald tests were used for comparisons of hygiene strictness. Strictness was estimated using proportional odds ordinal linear regression, adjusting for age, gender, and modern values.

Separate models were used for each domain.

**FIGURE 2:**
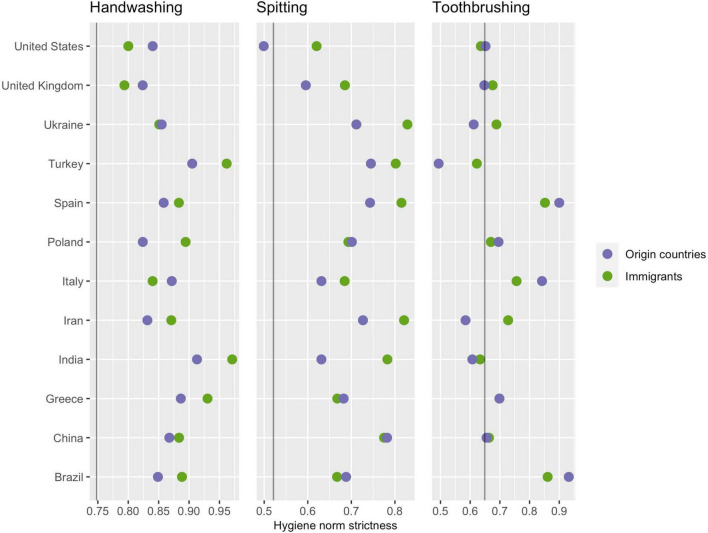
A country-level comparison of hygiene norm strictness. Means were estimated using proportional odds ordinal logistic regression, adjusting for age, gender, and modern values. The vertical lines represent hygiene strictness in Sweden.

The proportional odds ordinal logistic regression was also used to estimate the mean strictness for immigrants and nationals in each individual country, adjusting for age, gender, and modern values. See Figure 2, illustrating that results varied across countries, *X*^2^(*df* = 33) = 1676.7, *p* < 0.001, as well as across domains, *X*^2^(*df* = 24) = 67.8, *p* < 0.001; there is also an interaction between the country of origin and hygiene domain, *X*^2^(*df* = 22) = 54.2, *p* < 0.001. However, due to small immigrant samples per country, it is not meaningful to try to interpret specific country differences. In Figure 2, note that there are some exceptions to the overall finding that immigrants have stricter hygiene norms than both nationals and Swedes; for example, the handwashing strictness among immigrants from the United States is in between that of non-immigrant nationals and Swedes. But because the immigrant samples per country are small, we cannot say whether the exceptions are genuine or to sampling error, and note that no country was an exception in every domain.

## Discussion

We hypothesized that the strictness of hygiene norms among immigrants to Sweden would be somewhere in between that of Swedes and nationals in their countries of origin. The results showed hygiene norms in Sweden to be generally looser than in immigrants’ countries of origin. Thus, according to our hypothesis, immigrants would be expected to have stricter norms than Sweden but looser than nationals in their countries of origin. The results revealed an entirely different pattern. For toothbrushing, immigrants had stricter norms than Swedes but did not differ significantly from nationals in their countries of origin. For handwashing and spitting, immigrants not only had stricter norms than Swedes but also had significantly *stricter* norms than nationals in their countries of origin. This raises two questions. Why did material living conditions and perceived disease threat, acculturation, and selection effects not push strictness in the direction of Swedish norms? And why did strictness for handwashing and spitting instead move in the opposite direction?

The first question has several plausible answers. Perceptions of disease threat are subjective, and though Swedes perceive the threat of disease to be low, immigrants may maintain the perceptions acquired in their countries of origin. Assimilation and acculturation strategies involve identification with the host culture, but discrimination or a lack of frequent interactions with Swedes may prevent immigrants from identifying with Swedish culture (Virta et al., 2004; Berry et al., 2006; Taras et al., 2013). Immigrants may also view hygiene as more symbolic than instrumental or materialistic and opt for a separation strategy for hygiene norms and behaviors. Selection effects would arise if the average immigrant to Sweden differed from the average person in their countries of origin. We thought it likely that immigrants who choose to move to Sweden would be more similar to Swedes than their average compatriots. This may still be the case in other areas, but with regard to hygiene, the results reveal immigrants to be even less similar to Swedes.

This leads us to the second question, which presents more of a puzzle. Increasing cultural differences between immigrants and the majority culture do not fit with available models of acculturation. Selection effects are a possibility, but the consistency across immigrants from different countries points toward some more general mechanism. We propose a two-part explanation based on (1) a fundamental asymmetry in social sanctioning of violations of hygiene norms, such that being perceived as having too loose hygiene is more costly than being perceived as having too strict hygiene, and (2) immigrants are likely to be uncertain about prevailing hygiene norms in their new social environment.

The background to this explanation is a previous study on a “civilizing process” that gradually tightens shared standards of appropriate behaviors (Elias, 1978). It was recently proposed that the civilizing process is driven by the above-mentioned asymmetry in the propensity to sanction norm violations (Strimling et al., 2018). Looser hygiene behaviors may be seen as sloppy, negligent, and threatening, and evoke disgust and fear of infection (Curtis, 2007). Not living up to hygiene standards is thus more likely to provoke emotional responses from others, including sanctioning behaviors such as avoidance, gossip, or confrontation (Fehr and Gächter, 2002; Yamagishi et al., 2009; Eriksson and Strimling, 2020). Aggregated over many everyday interactions, the greater propensity to sanction loose behaviors can shift hygiene norms toward those who prefer stricter standards (Strimling et al., 2018). In a new social environment, it can be unclear what the local norms are for socially appropriate behavior. With imperfect knowledge of actual norms, people must act on their perceptions about which behaviors are most in their interest. Against the backdrop of the higher social cost of being perceived as unhygienic, it could then be rational to adopt *extra* strict hygiene.

This reasoning may apply doubly to immigrants. First, immigrants already risk being viewed as an out-group and face prejudice and stigmatizing stereotypes. Second, poor hygiene, disgust, and perceived disease threat have been shown to be associated with xenophobia and in-group bias (Faulkner et al., 2004; Schaller and Neuberg, 2012; Zakrzewska et al., 2019; Millar et al., 2020). Social sanctions are more likely for more visible norm transgressions. Consistent with this, immigrant hygiene norms were only significantly stricter for handwashing and spitting, which are more visible and public, and not for toothbrushing, which is less visible and more private. However, the null results for toothbrushing may also be explained by ceiling effects—it is rare in most countries to brush your teeth more than two times a day—or by differences in the measurement scale; For toothbrushing, there were only three response options, while strictness of handwashing and spitting was based on judgments about six different situations.

A possible alternative explanation is that the stricter hygiene norms are a reaction to more general acculturative stress. Immersion into a new social and cultural environment involves many challenges, such as learning a new language and brokering cultural differences, often leading to feelings of alienation, identity confusion, and a loss of control (Berry et al., 1987; Berry, 2006). Much research has shown that having an internal locus of control, that is, believing that one has control over outcomes in one’s life, facilitates effective coping with stressful events (Krause and Stryker, 1984; NG et al., 2006; Karstoft et al., 2015). In migrant samples, an internal locus of control has been shown to buffer the negative effects of perceived stress on mental health and life satisfaction (Young, 2001; Xia and Ma, 2020). Thus, in a situation where many things seem to be outside of their control, immigrants may adopt stricter hygiene norms to maintain a positive self-concept and a sense of control over their lives. However, in the literature on coping with acculturative stress, locus of control is mainly considered a personality characteristic rather than a coping strategy (Kuo, 2014). Further, there does not appear to be any research indicating that taking control over some aspects of life to compensate for a general loss of control is a common response to acculturative stress.

Having discussed upstream explanations, it is also worth touching on possible downstream consequences of stricter hygiene norms among immigrants. Researchers have often taken an interest in hygiene norms because of the relationship between poor hygiene and the spread of infectious diseases (Whitby et al., 2007; Curtis et al., 2009). Seemingly consistent with preferences for stricter hygiene, immigrants tend to have better health outcomes than natives in both their countries of origin and their destination countries. Dubbed the *healthy immigrant effect*, this phenomenon has been observed in many countries, (Kennedy et al., 2015; Farré, 2016; Vang et al., 2017; Markides and Rote, 2019). The effect is typically attributed to a positive selection of health characteristics, but it is possible that selection for healthy immigrants also selects for immigrants with stricter hygiene. However, the effect has mainly been found for chronic and lifestyle-associated diseases and tends to decrease over time, which seems to indicate that it has more to do with health status at the time of arrival than with stable personality traits or hygiene preferences that may protect against infection (Newbold, 2006; Gushulak, 2007; Elshahat et al., 2021). Nonetheless, future studies could further investigate possible connections between stricter hygiene norms and immigrant health outcomes.

One possible limitation of the present study is that the samples were dominated by highly educated participants. That hygiene norms may vary with higher education is a legitimate concern, but in an additional analysis, comparing student and non-student samples, we found no effect of higher education on strictness in any of the three hygiene domains. As is common with studies based on hard-to-survey populations, another potential limitation is our ability to generalize the results to all newly arrived immigrants. Individuals who lack literacy or digital competency, or have a low trust toward survey research, did not answer our study and could potentially have other hygiene norms than our respondents. Still, there is little reason to assume that these factors systematically bias the comparisons. For one thing, these factors are likely correlated with educational level, which does not appear to impact hygiene strictness. For another, the samples of nationals, which were dominated by students, would also be biased against individuals with low trust or that lack literacy or digital competence.

The present study compared the strictness of hygiene norms of immigrants to Sweden with the strictness in Sweden and in their countries of origin. The stricter hygiene norms of immigrants demonstrate that acculturation patterns are more complicated than gradual assimilation and highlight that patterns of acculturation may depend on the specific social pressures that surround different norms and social conventions. For hygiene norms, we suggest that there may not be any social pressure to loosen norms, as exceeding accepted hygiene standards is unlikely to meet social sanctions. Furthermore, uncertainty about prevailing norms may explain why immigrants displayed even stricter hygiene norms than nationals in their countries of origin. We intend to conduct a follow-up study in a couple of years that may clarify these dynamics. On the one hand, without pressure to conform to looser hygiene norms, immigrants may continue to have stricter hygiene norms than the broader Swedish population. On the other hand, if uncertainty about hygiene norms is a major factor, immigrant hygiene norms can be expected to gradually loosen as immigrants become more familiar with the norms and expectations of the majority culture.

## Data availability statement

The raw data supporting the conclusions of this article will be made available by the authors, without undue reservation.

## Ethics statement

The studies involving human participants were reviewed and approved by Swedish Ethical Review Authority. The patients/participants provided their written informed consent to participate in this study.

## Author contributions

KE and PS conceived and designed the study. JK did the literature review and drafted the manuscript. AT performed the data collection and wrote the application for ethical review. IH performed the statistical analysis. All authors gave critical input and approved the manuscript.

## Footnotes

1
https://aspredicted.org/blind.php?x=qy6et2
