# Supplementary figures and images for: Acculturation of hygiene norms among immigrants to Sweden
Source: Front Psychol. 2023 Feb 6;14:975361. doi: 10.3389/fpsyg.2023.975361 (PMC9939453; doi:10.3389/fpsyg.2023.975361)

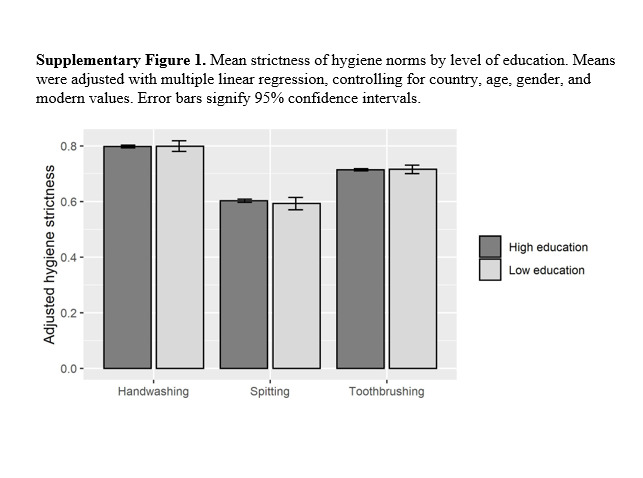

Supplement: Supplementary file 1 [file Image_1.JPEG]

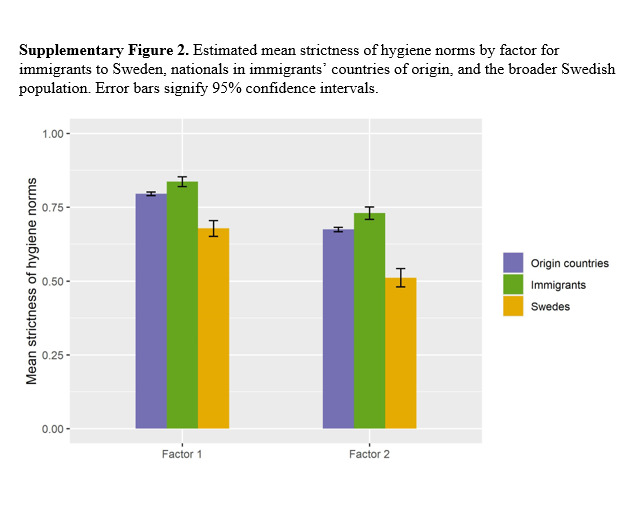

Supplement: Supplementary file 2 [file Image_2.JPEG]

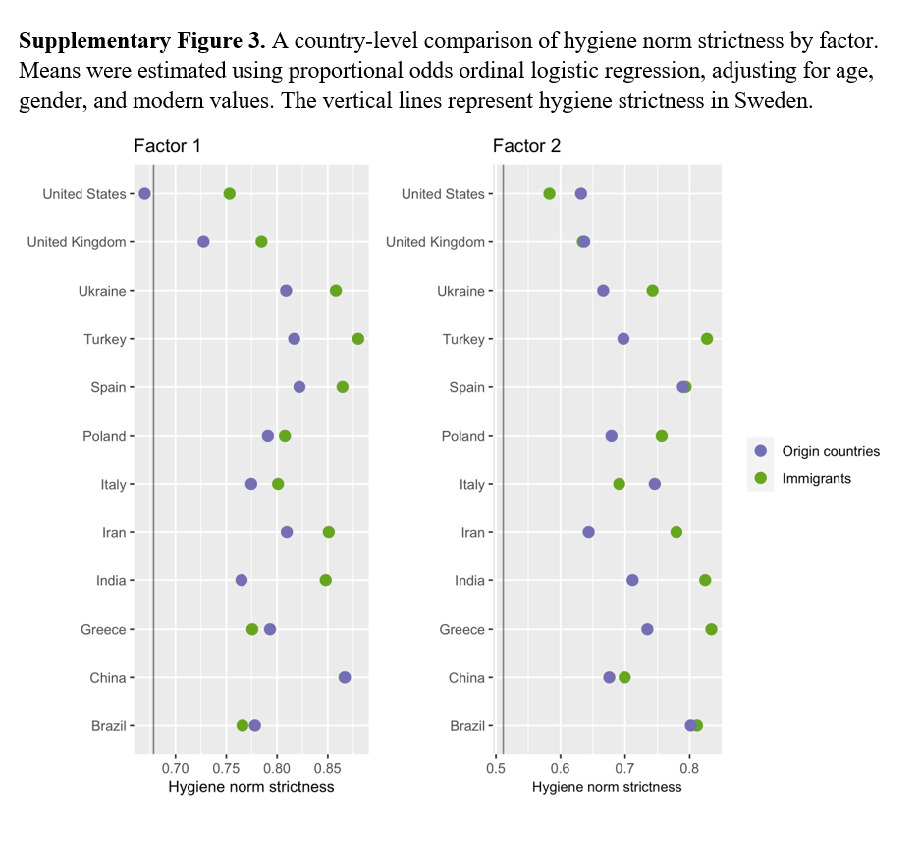

Supplement: Supplementary file 3 [file Image_3.jpg]

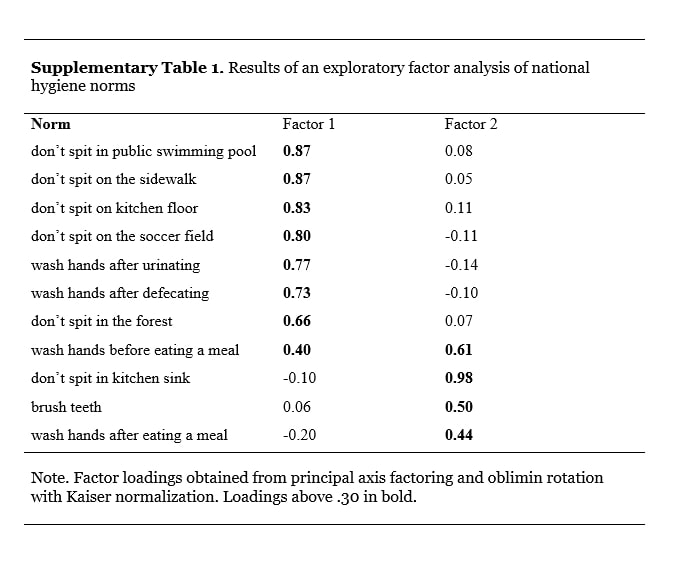

Supplement: Supplementary file 4 [file Image_4.JPEG]

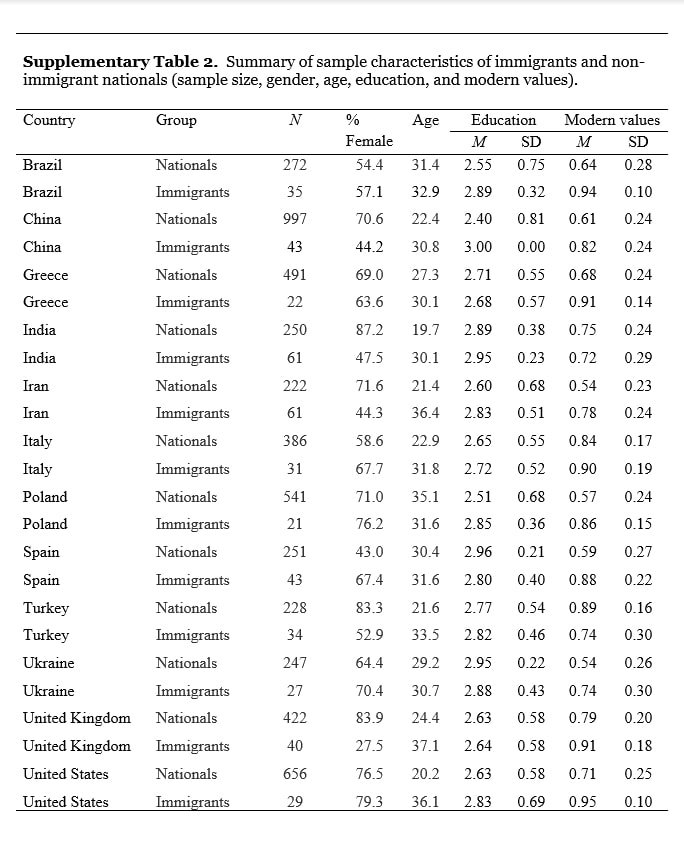

Supplement: Supplementary file 5 [file Image_5.JPEG]
